# ALL classification using neural ensemble and memetic deep feature optimization

**DOI:** 10.3389/frai.2024.1351942

**Published:** 2024-04-09

**Authors:** Muhammad Awais, Riaz Ahmad, Nabeela Kausar, Ahmed Ibrahim Alzahrani, Nasser Alalwan, Anum Masood

**Affiliations:** ^1^Department of Electrical and Computer Engineering, COMSATS University Islamabad, Wah, Pakistan; ^2^Department of Computer Engineering, TED University Ankara, Ankara, Türkiye; ^3^Department of Computer Science, Iqra University Islamabad, Islamabad, Pakistan; ^4^Department of Computer Science, COMSATS University Islamabad, Wah, Pakistan; ^5^Department of Computer Science, Community College, King Saud University, Riyadh, Saudi Arabia; ^6^Department of Physics, Norwegian University of Science and Technology, Trondheim, Norway; ^7^Department of Radiology, Boston Children's Hospital, Boston, MA, United States

**Keywords:** deep neural networks, optimization, meta-heuristics, transfer learning, convolutional neural network

## Abstract

Acute lymphoblastic leukemia (ALL) is a fatal blood disorder characterized by the excessive proliferation of immature white blood cells, originating in the bone marrow. An effective prognosis and treatment of ALL calls for its accurate and timely detection. Deep convolutional neural networks (CNNs) have shown promising results in digital pathology. However, they face challenges in classifying different subtypes of leukemia due to their subtle morphological differences. This study proposes an improved pipeline for binary detection and sub-type classification of ALL from blood smear images. At first, a customized, 88 layers deep CNN is proposed and trained using transfer learning along with GoogleNet CNN to create an ensemble of features. Furthermore, this study models the feature selection problem as a combinatorial optimization problem and proposes a memetic version of binary whale optimization algorithm, incorporating Differential Evolution-based local search method to enhance the exploration and exploitation of feature search space. The proposed approach is validated using publicly available standard datasets containing peripheral blood smear images of various classes of ALL. An overall best average accuracy of 99.15% is achieved for binary classification of ALL with an 85% decrease in the feature vector, together with 99% precision and 98.8% sensitivity. For B-ALL sub-type classification, the best accuracy of 98.69% is attained with 98.7% precision and 99.57% specificity. The proposed methodology shows better performance metrics as compared with several existing studies.

## 1 Introduction

Blood is an essential element for life and general health of human beings. It performs several crucial functions including transport of nutrients and waste materials, controlling flow of oxygen and overall immune system of body. Human blood is composed of three main types of blood cells, namely, erythrocytes, thrombocytes, and leukocytes. Each cell type performs a specific function in the human body. For example, leukocytes also referred as white blood cells (WBCs) are responsible for human immune and inflammatory response against diseases. Any abnormality in the structure and count of blood cells leads to certain diseases. As an example, leukemia, a blood malignancy, is caused due to an excessive leukocyte production in the bone marrows.

Leukemia is a widespread disease with over 475,000 new cases diagnosed worldwide each year and 312,000 annual deaths (Sung et al., 2021). With 62,770 new cases and 23,670 deaths anticipated, leukemia remains a significant public health concern for the United States in 2024 (Siegel et al., 2024). It is primarily categorized into two types: acute and chronic. Acute leukemia is distinguished by the rapid and unregulated proliferation of immature white blood cells within the bone marrow, which displaces the healthy cells. The fast progression of disease requires prompt response. On the other hand, the chronic leukemia is a slow progressing disease in which gradual accumulation of mature but abnormal WBCs takes place. Although these cells are typically more functional than those found in acute leukemia, they are aberrant and can still affect the normal functionality of blood and bone marrow. The acute and chronic categories of leukemia are further classified into myeloid and lymphoblastic sub-types, based on their afflicted cells. The acute lymphoblastic type of leukemia (ALL) affects the lymphoid cells and has high likelihood of occurring in the children and young adults. It represents ~14% of all new leukemia cases. Approximately 90% of ALL cases occur in individuals younger than 20 years old, with a peak incidence observed in children aged 2–5 (Sung et al., 2021). An estimated 6,550 new cases of ALL are expected in the US in 2024 (Siegel et al., 2024).

A form of acute lymphoblastic leukemia called B-cell acute lymphoblastic leukemia (B-ALL) develops from abnormal B-cell progenitors. Various sub types of B-ALL are further categorized based on distinct genetic, molecular, and immunophenotypic characteristics. Sub types of B-ALL include pre-cursor, mature, common, and pro B cell all.

The classical approach for the diagnosis of leukemia involves visual analysis of microscopic blood images by hematologists. This manual process needs human supervision; therefore, it is a time-consuming process and often prone to classification errors due to several factors (Matek et al., 2019). Thus, an accurate, computer-aided diagnosis of leukemia is highly desirable (Khattak et al., 2022). Among the modern approaches of computer vision, deep CNNs have demonstrated significant potential for a number of classification tasks in the biomedical domain. However, the computer vision-based blood analysis for leukemia diagnosis is difficult due to the small size, irregular structure, and physical similarities across various blood components (Kassani et al., 2019). Moreover, the performance of CNNs depends heavily on their depth and structure. To obtain a high level of accuracy requires a large, accurately labeled dataset for deep neural network training from the scratch. However, due to a number of limitations, such datasets are frequently not easily accessible in the biomedical domain. In such a context, transfer learning stands out as the recommended strategy, entailing the retraining of a deep CNN originally trained on a substantially extensive dataset to suit a specific classification task. A number of pretrained CNNs have achieved high top-1 accuracy on benchmark datasets. GoogleNet (Szegedy et al., 2015), Resnet (He et al., 2016), Darknet (Redmon and Farhadi, 2018), Densenet (Howard et al., 2017), and Inception (Chollet, 2017) are a few to mention. Recent research uses deep CNNs as extractors of features, which are then utilized to train outer classifiers. This leverages the power of transfer learning, allows for task-specific adaptation, and provides an efficient way to build accurate models. However, due to a large number of layers, deep CNNs extract high dimensionality feature representations from the input data. Afterward, feature selection is done to reduce the dimensionality of these extracted features, making them more manageable and potentially more informative. Efforts in current research are directed toward optimizing the computational efficiency and memory demands of the classification pipeline. The primary goal is to attain superior accuracy while operating with a more streamlined feature set (Khan et al., 2020; Ahmad et al., 2023b).

The remainder of the study is structured as follows: Section 2 presents a literature review of some recently published studies in the domain of leukemia identification. Section 3 offers an elaborate exposition of the proposed framework for ALL identification. In Section 4, we present and analyze simulation results, while discussion is concluded in Section 5.

## 2 Literature review

Table 1 presents a summary of some notable contributions in the realm of lekuemia identification using deep learning. They are discussed as follows. In the study mentioned in the reference, Elhassan et al. (2022), an approach is proposed for the detection of acute myeloid leukemia (AML) from WBC images. At first, a CMYK moment-based localization method is proposed to isolate the region of interest (ROI) from WBC images. This is followed by extraction and fusion of several pointwise and spatial features. Classification is performed using multiple classifiers including SVM and XG boost. The study reports the best accuracy of 97.57% on self collected single cell morphological dataset. In the study mentioned in the reference, Dese et al. (2021), a computer-assisted system is proposed for the diagnosis of several leukemia sub-types. The system is based on Gaussian and Weiner filtering for image pre-processing, followed by K-means clustering and marker-controlled Watershed algorithm for segmentation. Several morphological, texture, and statistical features are extracted and classified using multi-class SVM classifier. The best accuracy of 97.69% is reported for overall leukemia detection on self-collected dataset of peripheral blood smear images. In Al-jaboriy et al. (2019), an automatic method for the diagnosis of leukemia is proposed based on leukocyte cell segmentation. The method uses a dataset of 108 microscopic images and performs ANN-based segmentation and extracts various statistical features for classification. The best accuracy of 96% is achieved for binary classification of leukocyte cell blasts. The study mentioned in the reference, Kassani et al. (2019), the authors applied different augmentation techniques to the dataset images. Then, a hybrid CNN model consisting of hidden layers of VGG16, and MobileNet is proposed for feature extraction. The extracted features are classified using a NN architecture. The proposed method achieves a binary classification accuracy of 96.17%. In the study mentioned in the reference Jung et al. (2022), the authors proposed a custom CNN model for WBC classification for leukemia detection. The authors first created a synthetic dataset of WBC images using generative adversarial networks and then performed transfer learning of the proposed CNN for classification. An average accuracy of 97% is achieved by the system.

**Table 1:** Summary of some published studies on leukemia identification.

**Work**	**Year**	**Methodology**	**Leukemia type**	**Results**
Batool and Byun (2023)	2023	Data augmentation	ALL	Binary accuracy = 99.31%
		Classification: EfficientNetB3		Multiclass accuracy = 96.81%
Elhassan et al. (2022)	2022	CMYK based ROI localization	AML	Accuracy = 97.57%
		Feature extraction: pointwise, spatial features		
		Classification: SVM, XG boost		
Dese et al. (2021)	2021	Preprocessing: median and Wiener filter	ALL, AML,	Accuracy = 97.6%
		Segmentation: K-means clustering, watershed algorithm	CLL, CML	
		Feature extraction: morphological, texture, statistical		
		Classification: multiclass SVM		
Kumar et al. (2020)	2020	Feature extraction: K-best algorithm	ALL, AML	Accuracy = 97.25%
		Classification: SVM, random forest, DT		
Al-jaboriy et al. (2019)	2019	Segmentation: AI based	ALL	Accuracy = 96%
		Statistical feature extraction		
		Classification: ANN		
Kassani et al. (2019)	2019	Multiple augmentation techniques	ALL	Accuracy = 96.17%
		Classification: hybrid CNN model		Sensitivity = 95.17%

For extracting and choosing blood features, the authors of the study mentioned in the reference, Alruwaili (2021) presented a stepwise linear discriminant analysis technique. The suggested method performs the identification of specific attributes within blood smear images and their classification based on partial *F*-values. A Matlab-based method for classifying and identifying WBC cancer was proposed in the study mentioned in the reference, Nithyaa et al. (2021). The approach integrates a range of morphological, clustering, and image pre-processing procedures with the utilization of random forest classification. In the study mentioned in the reference, Pang et al. (2015), an automatic leukocyte categorization approach is proposed. Initially, moment invariants are derived using the Euclidean distance transform within the nucleus region, followed by the extraction of morphological characteristics from the segmented cells.

The published literature on leukemia detection also proposes a number of proprietary deep CNNs and their ensembles. In the study mentioned in the reference, Batool and Byun (2023), a lightweight deep learning-based EfficientNet-B3 model is proposed which employs depth-wise separable convolutions for ALL classification. The method proposed in this study attains a classification accuracy of 96.81% when applied to publicly available datasets for leukemia sub-type classification. In the study mentioned in the reference, Kumar et al. (2020), a simple method for the detection of ALL, and AML is proposed in which KBest algorithm is used for feature extraction, followed by a dense CNN for classification. The proposed approach reports the best accuracy of 97.2%. In the study mentioned in the reference, Jha et al. (2022), a leukemia identification method is proposed which uses K-means clustering from image segmentation. Next, multiple statistical features are extracted to train an ensemble of multiple classifiers. The proposed system reports a best accuracy of 96.3%. In most of the existing studies that utilize deep transfer learning, the feature selection is performed using a filter or wrapper-based approach. Filter-based methods assess the relevance of individual features by examining their statistical properties, such as correlation with the target variable or variance within the feature. These methods have a limitation in that they do not consider the relevance between the selected features and the actual model's performance. This can lead to situations where selected features might not be the most predictive for the planned model. Conversely, wrapper-based methods entail employing a machine learning model in the capacity of a “wrapper” to assess the effectiveness of various feature subsets. These methods select features by repeatedly training and evaluating the model on different subsets of features. These methods are particularly useful in obtaining the best set of features for a specific classifier model. Recently, population-based algorithms for feature selection have received considerable research attention. A significant challenge lies in fine tuning the algorithm to achieve better exploration of feature search space and obtain the most discriminant and powerful set of features. Standard population-based algorithms used in several studies on disease classification often suffer from poor convergence and local optima problems (Gupta et al., 2020; Shahzad et al., 2022).

### 2.1 Contributions

In this study, a hybrid method is proposed for the classification of ALL sub-types. The key contributions of this research can be outlined as follows:

- First, we present a customized 88-layer deep CNN architecture which incorporates the aspects of two standard deep CNN models, namely, AlexNet and SqueezeNet.
- Subsequently, we employ transfer learning to extract features using the proposed custom CNN architecture and another deep model, namely, GoogleNet. The feature vectors from both networks are fused together.
- For feature selection, we propose a memetic algorithm which combines a nature inspired meta-heuristic, i.e., whale optimization algorithm (WOA) with local search based on differential evolution. The proposed method achieves a better exploration of search space while avoiding local optima.
- The set of selected features is then used to perform training and classification using several outer classifiers with multiple kernel settings.
- The proposed pipeline is validated using public datasets for binary detection and sub-type classification of ALL. Better or comparable performance with significant reduction in feature vector size is demonstrated by the proposed method as compared with several existing studies.

## 3 Materials and methods

### 3.1 Datasets

**Figure 1:**
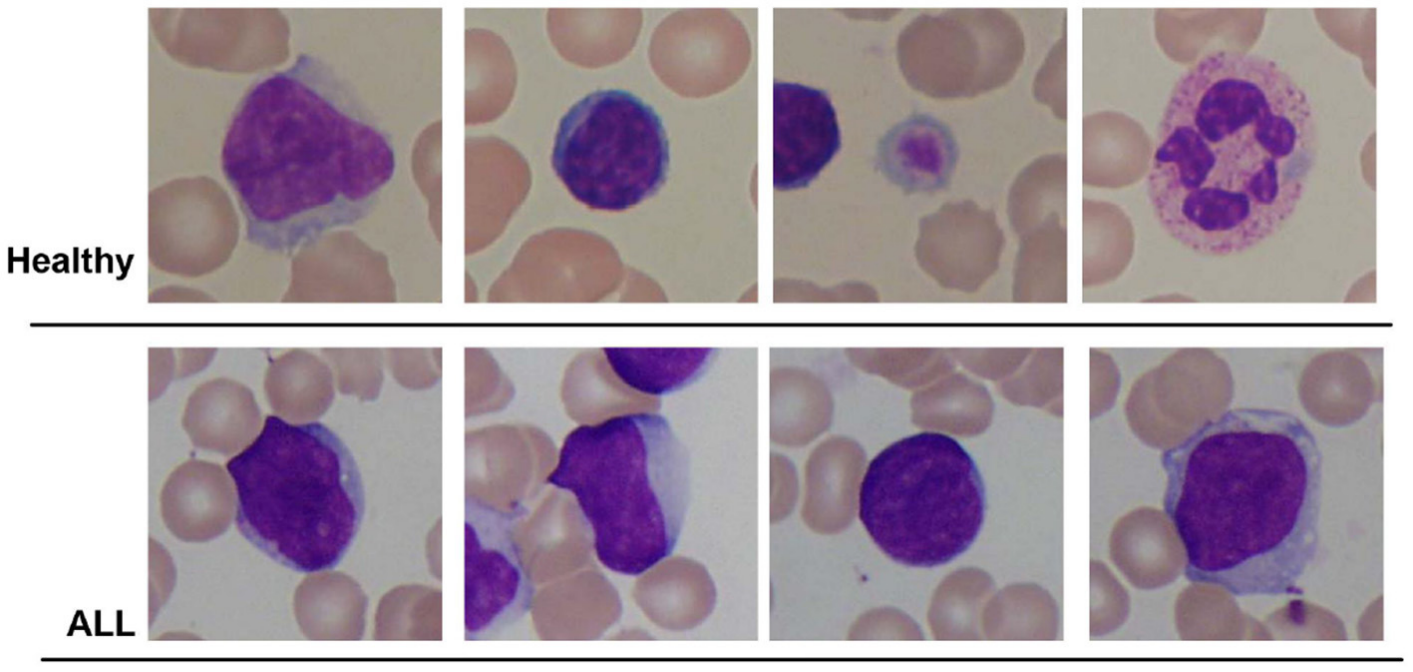
Sample images of ALL-IDB2 dataset of the study mentioned in the reference, Scotti et al. (2005).

In this research, publicly accessible datasets comprising blood smear images are employed for both binary detection and the identification of ALL sub-types. The first dataset is the ALL-IDB2 dataset created by the authors of the study mentioned in the reference, Scotti et al. (2005) at the University of Milan. This dataset consists of 260 images corresponding to two classes of subjects, i.e., “Healthy” and “ALL.” An optical microscope with a Canon Power Shot G5 camera is used to capture the images. The ALL-IDB2 dataset consists of cropped images of ALL-IDB1 dataset that obtains region of interest of normal and blast cells. The image resolution is 2, 592 × 1, 944 pixels with a TIFF format. Few samples of ALL-IDB2 dataset are shown in Figure 1.

**Figure 2:**
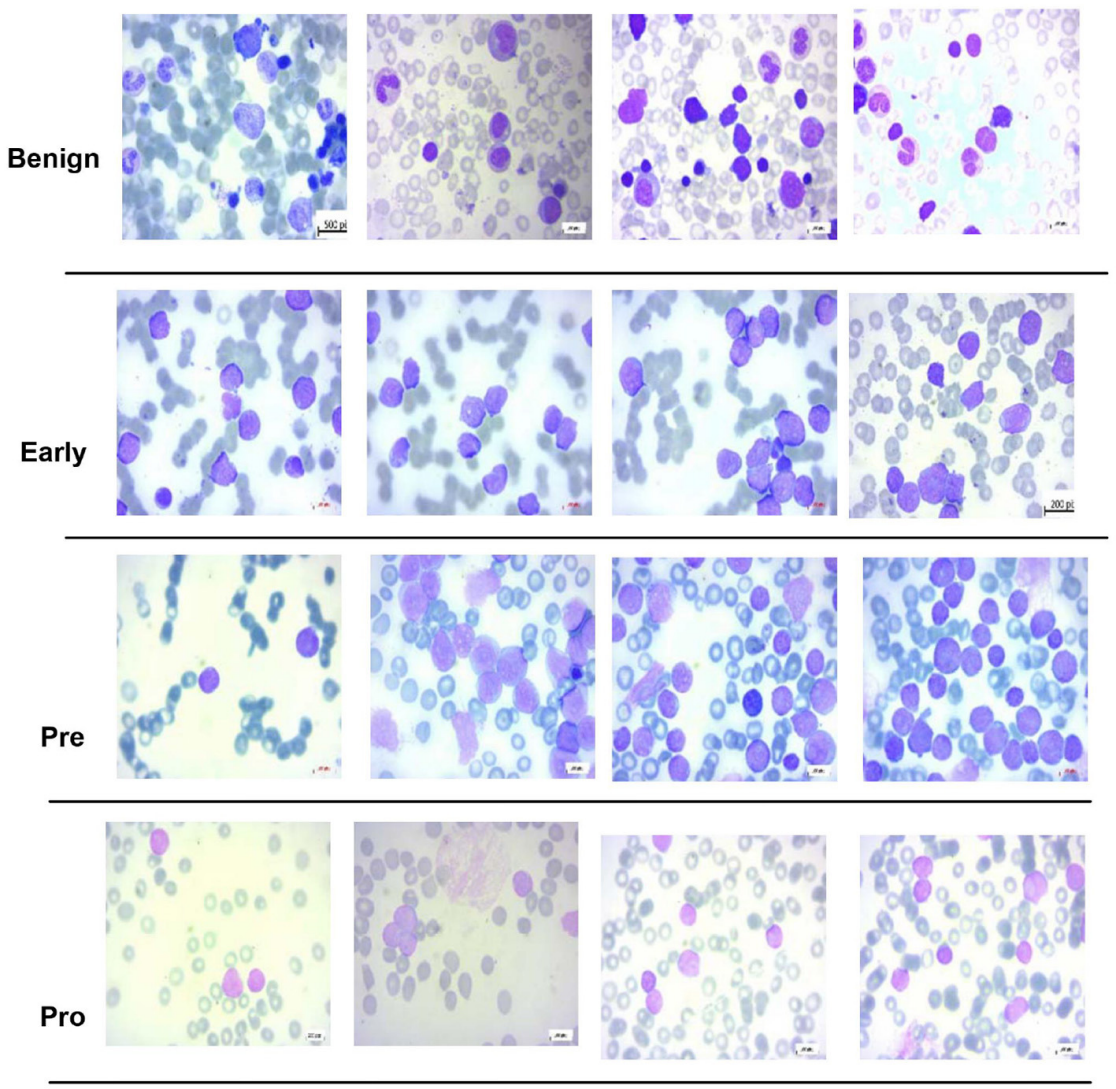
Sample images of dataset of the study mentioned in the reference, Ghaderzadeh et al. (2022).

For multi-class classification, this study uses the dataset of the study mentioned in the reference, Ghaderzadeh et al. (2022), which is prepared at bone marrow laboratory of Taleqani Hospital Iran. The dataset is composed of 3, 242 images which are divided into “Benign” class and three sub types of B-Cell ALL, namely, “Early,” “Pre-cursor,” and “Pro B,” with a class distribution of 512, 955, 796, and 979 images, respectively. A microscope with 100× magnification of Zeiss Camera is used to capture the images having 224 × 224 pixel resolution. Few images of this dataset are shown in Figure 2.

### 3.2 Computation pipeline

**Figure 3:**
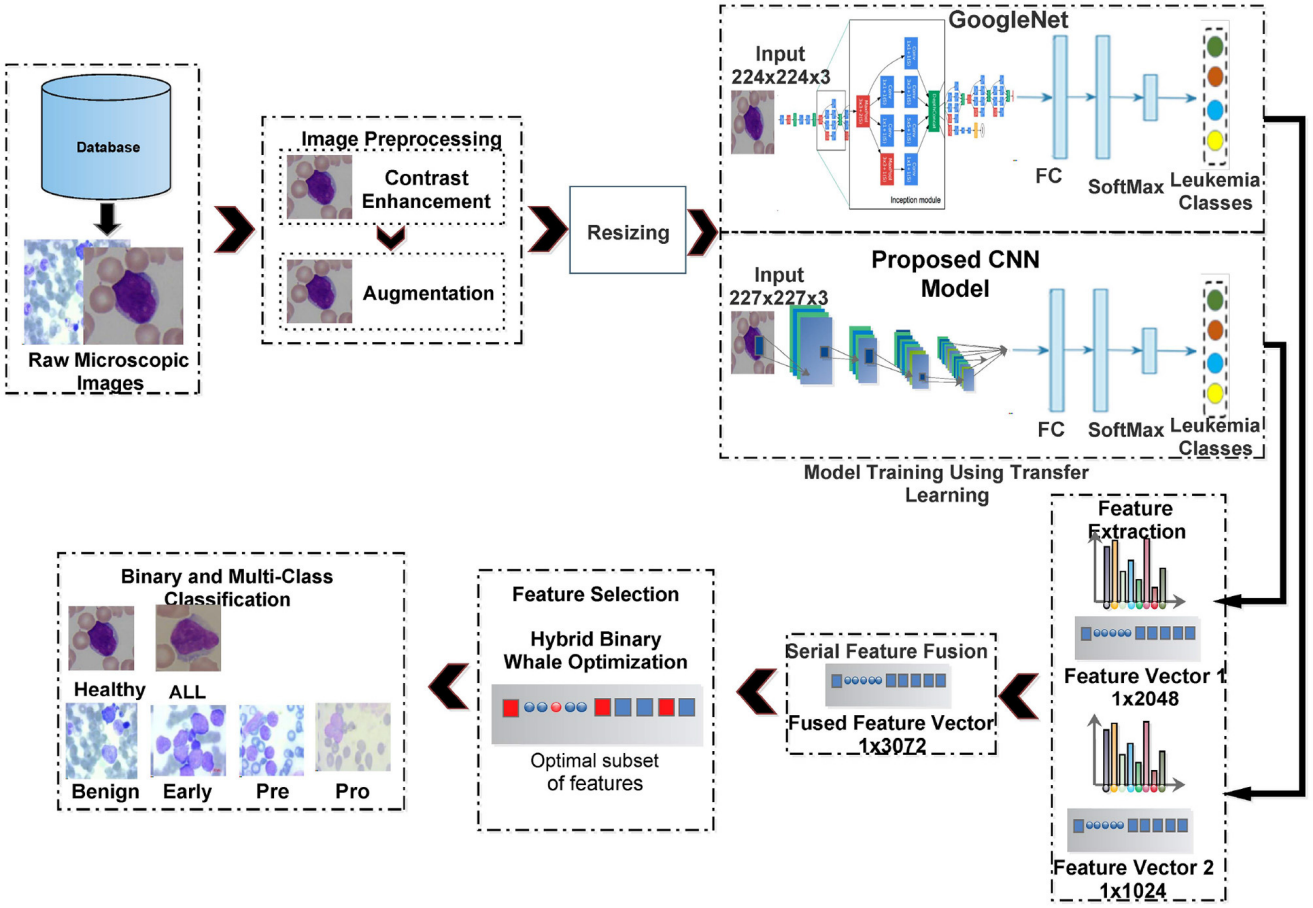
Proposed framework for binary and sub-type classification of ALL.

Figure 3 shows the computation pipeline of the proposed framework for ALL identification and its sub-type classification. The pipeline accepts the raw microscopic images from selected database repositories. These images are then pre-processed using contrast enhancement and augmentation steps. The contrast-enhanced images are resized according to input layer requirements of two deep neural networks, i.e., GoogleNet and our proposed CNN and subjected to transfer learning step. The features extracted from these deep CNNs are serially fused together and then subjected to the feature selection step. The selected set of features is then classified using multiple classifiers. These steps are discussed in details as follows.

#### 3.2.1 Dataset pre-processing

In the first step, the training and testing dataset images are subjected to contrast enhancement using color histogram equalization. When dealing with microscopic images, contrast enhancement by applying histogram equalization independently to R, G, and B channels may not always produce good results (Xie et al., 2019). Equalizing the histogram across all three RGB channels can amplify existing noise in the image, especially in areas with low intensity values. This can make it difficult to distinguish between relevant features and noise artifacts. This study performs image contrast enhancement within the HSI image domain. HSI separates intensity information from hue and saturation, making it less susceptible to variations in lighting conditions that can affect RGB channels. This is particularly helpful for microscopic images, where lighting control can be challenging. By separating hue, saturation, and intensity, HSI provides distinct channels that can be individually analyzed or combined to extract specific features relevant to the recognition task. This can improve the ability to differentiate between different cell types, structures, or objects in the image.

The main steps of image contrast enhancement adopted in this study are as follows:

1. Transform the RGB image into the HSI image;
2. Perform histogram equalization on the intensity channel;
3. Substitute the HSI image's intensity channel with the corresponding histogram-equalized intensity channel;
4. Revert the HSI image back to an RGB image.

#### 3.2.2 Customized deep feature extraction

Feature extraction stands as a pivotal phase within the domain of deep learning. In this study, we employ transfer learning from a standard deep CNN, i.e., GoogleNet and our proposed custom CNN architecture for feature extraction. Both of these networks are elaborated upon as follows.

##### 3.2.2.1 GoogleNet

GoogleNet also referred to as InceptionV1 is a deep CNN architecture developed by the researchers at Google (Szegedy et al., 2015). It is designed to solve some problems of earlier networks such as vanishing gradient problem and trade-off between complexity and efficiency. To solve the problem of overfitting due to very deep neural networks, the GoogleNet is based on the idea of having multiple sized filters, operating in the same level. The resultant network becomes wider rather than becoming deeper. Breakthrough performance is achieved due to the introduction of ‘Inception modules” and auxiliary classifiers. An inception module is composed of parallel concatenation of convolutions with multiple sized kernels and pooling operations in order to allow efficient learning of local and global features. The GoogleNet also utilizes 1 × 1 convolutions which is also known as “network-in-network” layers. Incorporation of these layers before applying larger filter convolution results in a compact, computationally efficient network. Moreover, these layers are used to combine features across different inception modules for multi-abstraction feature learning.

The GoogleNet Architecture has 22 layers including nine linearly stacked inception modules, four max pool layers, a dropout regularization layer, and fully connected layer. The inception module terminations are linked to the global average pooling layer. The GoogleNet is pretrained on the ImageNet dataset,^1^ which consists of thousands of image categories. To facilitate transfer learning on the leukemia dataset, several modifications are made to the network. First, the last learnable layer, referred to as “loss3-classifier,” is substituted with a new fully connected layer having an output count and matching the number of leukemia classes. Additionally, the network's softmax layer is replaced with a new softmax layer. Furthermore, the classification layer of the network is substituted with a new classification layer without class labels. Before commencing training, dimensions of all images are changed to 224 × 224 × 3 to conform to the network's input layer. Subsequently, various augmentation techniques, such as flipping, scaling, and random rotation, are applied. The extraction of deep features is conducted from the global average pool layer, denoted as “pool5-7x7_s1,” which yields a deep feature vector comprising 1 × 1,024 features per image.

##### 3.2.2.2 Proposed custom network

**Figure 4:**
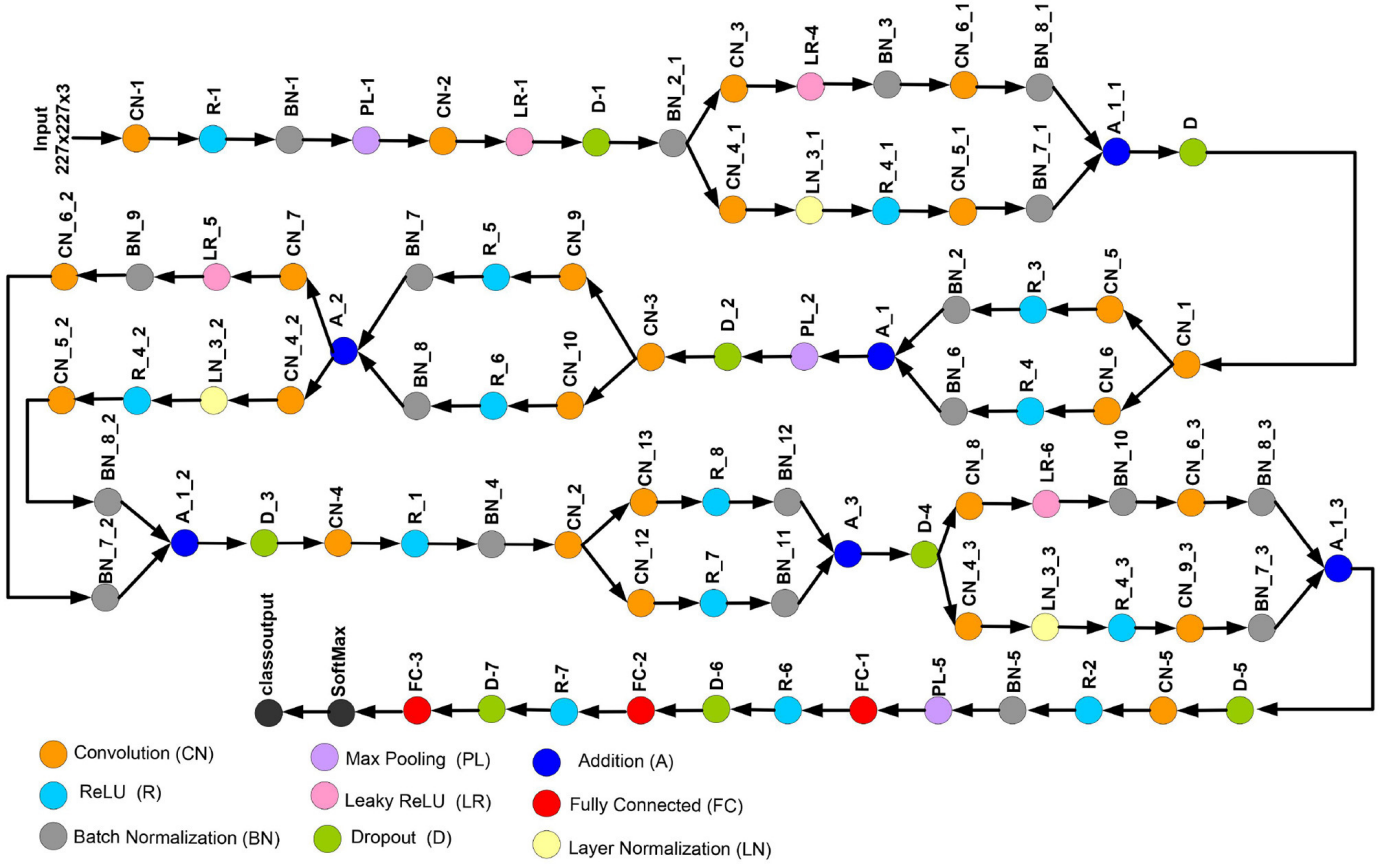
Proposed custom CNN architecture.

This study introduces a novel deep CNN, which is meticulously designed to incorporate key attributes from two well-known deep models: AlexNet and SqueezeNet. AlexNet is composed of five convolutional layers and three fully connected layers. Furthermore, it incorporates three pooling layers, seven ReLU activation layers, two dropout layers, and a SoftMax layer. In contrast, the proposed CNN model encompasses 88 layers, spanning from the input to the output layer. Beyond the conventional layers inspired by AlexNet, the proposed model introduces additional elements such as batch normalization and structures reminiscent of SqueezeNet. The architectural view of customized architecture is shown in Figure 4. The size of input layer is 227 × 227 × 3, which is similar to the AlexNet architecture. The network starts with a convolution (CN) layer followed by ReLU (R), Batch Normalization (BN), Max Poopling (PL), Leaky ReLU (LR), and Drop out (D) layers. Embedded in the network, are the SqueezeNet like structures of parallel branches of grouped convolution layers (having a cascade of CN, R, LR, A, and BN layers). The individual branches of each group are merged together with the help of Addition (A) layer. The last three layers of the network are fully connected (FC), softmax and classoutput layer. Tables 2, 3 present the detailed configuration of all layers of the proposed CNN architecture.

**Table 2:** Layer-specific details of the proposed CNN architecture.

**Layer#**	**Layer name**	**Filter map size**	**Filter depth**	**Stride**	**Padding**	**Learnable**
01	Input image	227 × 227 × 3	11 × 11 × 3 × 96	[4 4]	[0 0 0 0]	wt 227 × 227 × 3 B 1 × 1 × 96
02	CN1	55 × 55 × 96	–			
03	R1	55 × 55 × 96	–			
04	BN1	55 × 55 × 96	5 × 5 × 96 × 96	[1 1]	Same	Offset 1 × 1 × 256 scale 3 × 3 × 256
05	Pool	27 × 27 × 96	–			
06	CN2	55 × 55 × 256	–			wt 5 × 5 × 48 × 128 B 1 × 1 × 2 × 128
07	LR-1	27 × 27 × 256	1 × 1 × 96 × 48	[1 1]	Same	
08	Drop_1	27 × 27 × 256	–			
09	BN_2_1	27 × 27 × 256	11 × 11 × 48 × 96	[1 1]	Same	Offset 1 × 1 × 256 scale 3 × 3 × 256
10	LR-1	55 × 55 × 96				wt 5 × 5 × 48 × 128 B 1 × 1 × 2 × 128
11	LR-2	55 × 55 × 96				Offset 1 × 1 × 256 scale 3 × 3 × 256
12	ADD1	55 × 55 × 96				
13	CN-5	27 × 27 × 256				wt 5 × 5 × 48 × 128 B 1 × 1 × 2 × 128
14	BN_7_1	27 × 27 × 256		[1 1]	Same	Offset 1 × 1 × 256 scale 3 × 3 × 256
15	CN-3	27 × 27 × 256				wt 5 × 5 × 48 × 128 B 1 × 1 × 2 × 128
16	LR4	27 × 27 × 64				
17	BN3	27 × 27 × 64		[1 1]	Same	Offset 1 × 1 × 256 scale 3 × 3 × 256
18	CN_6_1	55 × 55 × 256				*wt*5 × 5 × 48 × 128 B 1 × 1 × 2 × 128
19	BN_8_1	27 × 27 × 64		[1 1]	Same	
20	Addition_1_1					
21	Dropout	13 × 13 × 384	3 × 3 × 256 × 384	[1 1]	[1 1 1 1]	
22	CN_1	55 × 55 × 256				wt 5 × 5 × 48 × 128 B 1 × 1 × 2 × 128
23	CN_6	55 × 55 × 256				wt 5 × 5 × 48 × 128 B 1 × 1 × 2 × 128
24	CN_5	55 × 55 × 256				wt 5 × 5 × 48 × 128 B × 1 × 2 × 128
25	Relu_3					
26	BN-2	27 × 27 × 64		[1 1]	Same	Offset 1 × 1 × 256 scale 3 × 3 × 256
27	Relu_4					
28	BN-6	27 × 27 × 64		[1 1]	Same	Offset 1 × 1 × 256 scale3 × 3 × 256
29	Addition_1					
30	pool-2	Max pool 3 × 3		[2 2]	[0 0 0 0]	
31	Dropout	13 × 13 × 256	13 × 13 × 256 × 384	[1 1]	[1 1 1 1]	
32	CN3	3 × 3 × 256				wt 5 × 5 × 48 × 128B 1 × 1 × 2 × 128
33	CN_10	3 × 3 × 256				wt 5 × 5 × 48 × 128 B 1 × 1 × 2 × 128
34	BN-6	13 × 13 × 64		[1 1]	Same	Offset 1 × 1 × 256 scale 3 × 3 × 256
35	CN_8	3 × 3 × 384				wt5 × 5 × 48 × 128 B 1 × 1 × 2 × 128
36	Relu_4					
37	BN-6	13 × 13 × 64		[1 1]	Same	Offset1 × 1 × 256 scale 3 × 3 × 256
38	Addition_2					
39	CN_4_2	55 × 55 × 256				wt 5 × 5 × 48 × 128 B 1 × 1 × 2 × 128
40	CN_7	55 × 55 × 256				
41	LR_5	13 × 13 × 64				wt 5 × 5 × 48 × 128 B × 1 × 2 × 128
42	BN-9	13 × 13 × 64				
43	LR_5	13 × 13 × 64		[1 1]	Same	Offset 1 × 1 × 256 scale 3 × 3 × 256
44	Relu_4					
45	CN_5_2	55 × 55 × 256				
46	BN_7_2	27 × 27 × 64				wt 5 × 5 × 48 × 128 B1 × 1 × 2 × 128
47	CN_6_2	55 × 55 × 256		[1 1]	Same	Offset 1 × 1 × 256 scale 3 × 3 × 256
48	BN_8_2	27 × 27 × 64				Offset 1 × 1 × 256 scale 3 × 3 × 256
49	Addition_1_2			[1 1]		
50	Dropout_3					
51	CN3	3 × 3 × 384				
52	Relu_1					

**Table 3:** Layer-specific details of the proposed CNN architecture (Contd.).

**Layer#**	**Layer name**	**Filter map size**	**Filter depth**	**Stride**	**Padding**	**Learnable**
**53**	**BN-4**	**13 × 13 × 384**				
54	CN_2	55 × 55 × 256		[1 1]	Same	wt 5 × 5 × 48 × 128 B 1 × 1 × 2 × 128
55	CN_12	55 × 55 × 256				wt 5 × 5 × 48 × 128 B 1 × 1 × 2 × 128
56	CN_13	55 × 55 × 256				wt 5 × 5 × 48 × 128 B 1 × 1 × 2 × 128
57	Relu_8					
58	BN-12	13 × 13 × 384				Offset 1 × 1 × 384 scale 3 × 3 × 384
59	Relu_7			[1 1]	Same	
60	BN-11	13 × 13 × 384				Offset 1 × 1 × 384 scale 3 × 3 × 384
61	Addition_3			[1 1]	Same	
62	Dropout_4					
63	CN_8	13 × 13 × 64				
64	CN_4_3	13 × 13 × 64				wt 5 × 5 × 48 × 128 B 1 × 1 × 2 × 128
65	LR_3_3	13 × 13 × 64				Offset 1 × 1 × 64 scale 1 × 31 × 64
66	Relu_4_3					
67	CN_5_3	13 × 13 × 384				wt 5 × 5 × 48 × 128 B 1 × 1 × 2 × 128
68	LR_6	13 × 13 × 64				
69	BN_10	13 × 13 × 64				Offset 1 × 1 × 64 scale 3 × 3 × 64
70	CN_6_3	13 × 13 × 384		[1 1]	Same	
71	BN_8_3	13 × 13 × 384				Offset 1 × 1 × 384 scale 3 × 3 × 384
72	BN_7_3	13 × 13 × 384		[1 1]	Same	Offset 1 × 1 × 384 scale 3 × 3 × 384
73	Addition_1_3			[1 1]	Same	
74	Dropout_5					
75	CN-5	13 × 13 × 256				wt 5 × 5 × 48 × 128 B 1 × 1 × 2 × 128
76	Relu_2					
77	BN-5	13 × 13 × 256				wt 5 × 5 × 48 × 128 B 1 × 1 × 2 × 128
78	Pool5			[1 1]	Same	
79	FC-1					
80	Relu6					
81	Drop6	1 × 1 × 2, 048				
82	FC-2					
83	Relu-7					
84	Drop7	1 × 1 × 2, 048				
85	FC-3					
86	SoftMax					
87	Output					

The leukemia datasets utilized in this research are relatively small, making it infeasible to train the proposed CNN model from the ground up. Consequently, the initial step involves pre-training the proposed CNN on the CIFAR-100 dataset (Krizhevsky et al., 2009), which encompasses 100 object categories, each with 600 images. Subsequently, transfer learning is applied to adapt the pre-trained network to the leukemia dataset. The extraction of deep features is conducted from the FC-3 layer, yielding a feature vector with dimensions of 1 × 2, 048 for each image.

#### 3.2.3 Feature ensemble/fusion

Obtained feature vectors from both networks are combined together through a serial concatenation technique. The joint feature vector has a size of 1 × 3, 072 features per image.

#### 3.2.4 Feature selection

Feature fusion enlarges the feature vector, potentially triggering the ‘curse of dimensionality' issue. This expanded feature vector may include duplicate features, which can result in overfitting by the classifier. Selection of the most relevant features is an essential step to achieve better generalization while reducing the computational complexity of the classification system. As an important contribution, this study models the problem of deep feature selection as a global combinatorial optimization problem and proposes a nature-inspired metaheuristic, i.e., whale optimization algorithm (WOA), to achieve the most pertinent set of features.

##### 3.2.4.1 Standard whale optimization algorithm

The WOA, as introduced by Mirjalili and Lewis in their study (Mirjalili and Lewis, 2016), offers a solution to the challenge of discovering optimal solutions within intricate search spaces. This algorithm emulates the social and hunting behaviors of humpback whales, leveraging their techniques to improve solutions within the search space. Humpback whales employ a bubble-net hunting strategy to corral and capture their prey, particularly in the case of small fish groups.

Mathematically, the algorithm begins with a random whale population. The optimization model captures three whale behaviors: (a) hunting for prey (exploration), (b) encircling the prey, and (c) executing a bubble-net attack (exploitation).

###### 3.2.4.1.1 Encircling the prey

The current best candidate solution of a population is called as the “leader.” It is the whale which has the best fitness value and assumed to be closest to the target prey. All other solutions (whales) update their position toward the leader. Mathematically, the position update is computed as follows (Mirjalili and Lewis, 2016):


(1) $$\begin{array}{c} \vec{Y}=|\vec{C}.\vec{X^{*}}\left(t\right)-\vec{X}\left(t\right)| \end{array}$$

(2) $$\begin{array}{c} \vec{X}\left(t+1\right)=\vec{X^{*}}\left(t\right)-\vec{A}.\vec{Y} \end{array}$$

where *t* denotes the current iteration number, $\vec{X^{*}}\left(t\right)$ is the leader, i.e., population best solution so far, $\vec{X}\left(t\right)$ is the individual whale. $\vec{A}$ and $\vec{C}$ are the co-efficient vectors calculated as follows (Mirjalili and Lewis, 2016):


(3) $$\begin{array}{c} \vec{A}=2.\vec{a}.\vec{r_{1}}-\vec{a} \end{array}$$

(4) $$\begin{array}{c} \vec{C}=2.\vec{r_{2}} \end{array}$$

where *r*_1_ and *r*_2_ are random numbers in [0, 1].

###### 3.2.4.1.2 Bubble-net attacking

This behavior of humpback whales is mathematically modeled using two approaches.

1. Shrinking encircle: to mimic this behavior, the value of $\vec{a}$ is decreased from 2 to 0 through a linear function (Mirjalili and Lewis, 2016)
  (5) $$\begin{array}{c} \vec{a}=2\left(1-\frac{t}{t_{max}}\right) \end{array}$$
  where *t*_*max*_ is the maximum number of iterations.
2. Spiral trajectory: the whales create an upward spiral loop around the prey. The position update due to this spiral trajectory is modeled as follows (Mirjalili and Lewis, 2016):
  (6) $$\begin{array}{c} \vec{Y}=|{\vec{X}}^{*}\left(t\right)-\vec{X}\left(t\right)| \end{array}$$
  (7) $$\begin{array}{c} \vec{X}\left(t+1\right)=\vec{Y}.e^{b.l}.cos\left(2.\pi .l\right)+\vec{X^{*}}\left(t\right) \end{array}$$
  where *l* is a random number in [−1, 1] and *b* is a constant.

The position update of whales considering both phenomenons of spiral trajectory and shrinking encirclement is performed as follows:


(8) $$\begin{array}{c} \vec{X}\left(t+1\right)=\{\begin{array}{ccc} \vec{X}\left(t\right)-\vec{A}.\vec{Y} & \; & p<0.5\\ \vec{Y}.e^{b.l}.cos\left(2.\pi .l\right)+\vec{X^{*}}\left(t\right) & \; & p\geq 0.5 \end{array}\} \end{array}$$

###### 3.2.4.1.3 Searching prey (exploration)

In addition to above hunting mechanisms, the humpback whales also search randomly according to position of each others. When $|\vec{A}|<1$, the position update of each whale is carried out using the Equation (1) whereas, for $|\vec{A}|\geq 1$, the position update is computed as follows (Mirjalili and Lewis, 2016):


(9) $$\begin{array}{c} \vec{Y}=|\vec{C}.{\vec{X}}_{r}\left(t\right)-\vec{X}\left(t\right)| \end{array}$$

(10) $$\begin{array}{c} \vec{X}\left(t+1\right)={\vec{X}}_{r}\left(t\right)-\vec{A}.\vec{Y} \end{array}$$

where *X*_*r*_(*t*) is the randomly selected whale as the population best solution.

##### 3.2.4.2 Proposed hybrid binary whale optimization algorithm

The optimal feature selection problem is a binary combinatorial optimization problem. Therefore, an association rule is required to convert the real valued whale position vectors into binary sub-space. In this study, we have proposed a “V”-shaped transfer function for whale position update as follows:


(11) $$\begin{array}{c} P\left({\vec{X}}_{ij}\right)=|\frac{2}{\pi }tan^{-1}\left(\frac{\pi }{2}{\vec{X}}_{ij}\right)| \end{array}$$

(12) $$\begin{array}{c} {\vec{X}}_{ij}=\{\begin{array}{cc} 1 & r_{1}\geq P\left({\vec{X}}_{ij}\right)\\ 0 & otherwise \end{array}\} \end{array}$$

where *r*_1_ denotes a uniformly distributed random number in [0, 1], and ${\vec{X}}_{ij}$ denotes the feature at index *j* of *i* − *th* whale.

In WOA, the whales update their position on the basis of optimal individual solutions (leader). Often, the algorithm may fall into the local optimum, resulting in a loss of population diversity. To avoid this problem, we have proposed a hybrid binary WOA, in which Differential Evolution (DE) is applied as a local search technique.

During each iteration of WOA, the so far best solution (leader) is computed. All other whales of the population update their position using the update rules (Equations 8, 11, 12). To perform local refinement of an optimum solution, the whole population of binary individuals is considered as an input to the DE algorithm which operates in the following steps.

###### 3.2.4.2.1 Mutation

Each individual (“target”) $\vec{X_{i}}$ in the population is used to generate its corresponding mutation vector $\vec{M_{i}}$ such that:


(13) $$\begin{array}{c} \vec{M_{i}}=\vec{X_{r1}}⊕\vec{X_{r2}} \end{array}$$

This mutation vector is then used to create a trial vector as follows:


(14) $$\begin{array}{c} \vec{U_{i}}=\vec{M_{i}}⊕\vec{X_{r3}} \end{array}$$

where $\vec{X_{r1}},\vec{X_{r2}}$, and $\vec{X_{r3}}$ are three randomly selected distinct vectors excluding $\vec{X_{i}}$, and ⊕ denotes the bit-wise XOR operation.

###### 3.2.4.2.2 Binomial crossover

The target vector $\vec{X_{i}}$ and trial vector $\vec{U_{i}}$ undergo the Binomial Crossover as follows:


(15) $$\begin{array}{c} \vec{C_{i,j}}=\{\begin{array}{cc} \vec{X_{ij}} & \text{if }x_{1}=j\text{ or }x_{2}\leq p_{r}\\ \vec{U_{ij}} & otherwise \end{array}\} \end{array}$$

where *j* = 1:*d*, *d* is the dimensionality of the *i*-th individual, *x*_1_ is a random number in interval [1, *d*], *x*_2_ is a random number in interval [0, 1], and *p*_*r*_ is the crossover probability.

Finally, the fitness of each cross-over individual $\vec{C_{i}}$ is computed. If there is an individual with fitness value better than the iteration best solution $\vec{X^{*}}$ of binary WOA, $\vec{X^{*}}$ is replaced by this individual.

###### 3.2.4.2.3 Feature selection using proposed hybrid binary WOA

Algorithm 1 shows the main computational steps of the proposed hybrid binary whale optimization (BWO)-based feature selection approach. Table 4 lists the main symbols and variables used in the algorithm. The algorithm receives the fused feature matrix 𝔽 of size *n*_*t*_ × *d*_*max*_, where *n*_*t*_ denotes the total number of images in the training set used for feature extraction, and *d*_*max*_ is the total number of fused features, i.e., 3, 072 per image. Each row of 𝔽 corresponds to fused feature vector obtained from a single image. *L* is a vector containing class labels of training dataset images, *t*_*max*_ is the maximum number of algorithm iterations, and *n*_*p*_ is the population size. In Step 4 of the algorithm, the whale population matrix $\vec{𝕏}$ of size *n*_*p*_ × *d*_*max*_ is randomly generated. The algorithm runs for *t*_*max*_ iterations. During each iteration, Steps 7–12 compute the fitness of each individual to update the best (leader whale) solution $\vec{X^{*}}$ and its fitness value Γ^*^. The fitness function *Evaluate* receives as input parameters the population matrix $\vec{𝕏}$, the label vector *L*, and one binary individual $\vec{X}$ of $\vec{𝕏}$. In Step 35, all features corresponding to non-zero entries of $\vec{X}$ are extracted from 𝔽 and stored in 𝔽_2_. In the subsequent Steps 36–38, the feature matrix 𝔽_2_ and label vector *V* are split into training and testing parts with holdout ratio of *h*_0_. Then, training of KNN classifier is performed, and predicted labels are obtained by applying testing feature set. The classification accuracy *a*_*c*_ and fitness Γ are computed as Equations 16, 17:


(16) $$\begin{array}{c} a_{c}=\frac{n_{pred}}{n_{test}}\times 100\left(\%\right) \end{array}$$

(17) $$\begin{array}{c} \Gamma =\alpha_{1}.\left(1-a_{c}\right)+\alpha_{2}.\frac{q_{s}}{q_{t}}; \end{array}$$

where *n*_*pred*_ and *n*_*test*_, respectively, denote the total number of successfully predicted and applied testing samples of KNN classifier. α_1_ and α_2_ are weight coefficients such that α_1_+α_2_ = 1. *q*_*s*_ and *q*_*t*_ denote the number of selected and total features of $\vec{𝕏}$.

**Algorithm 1:**
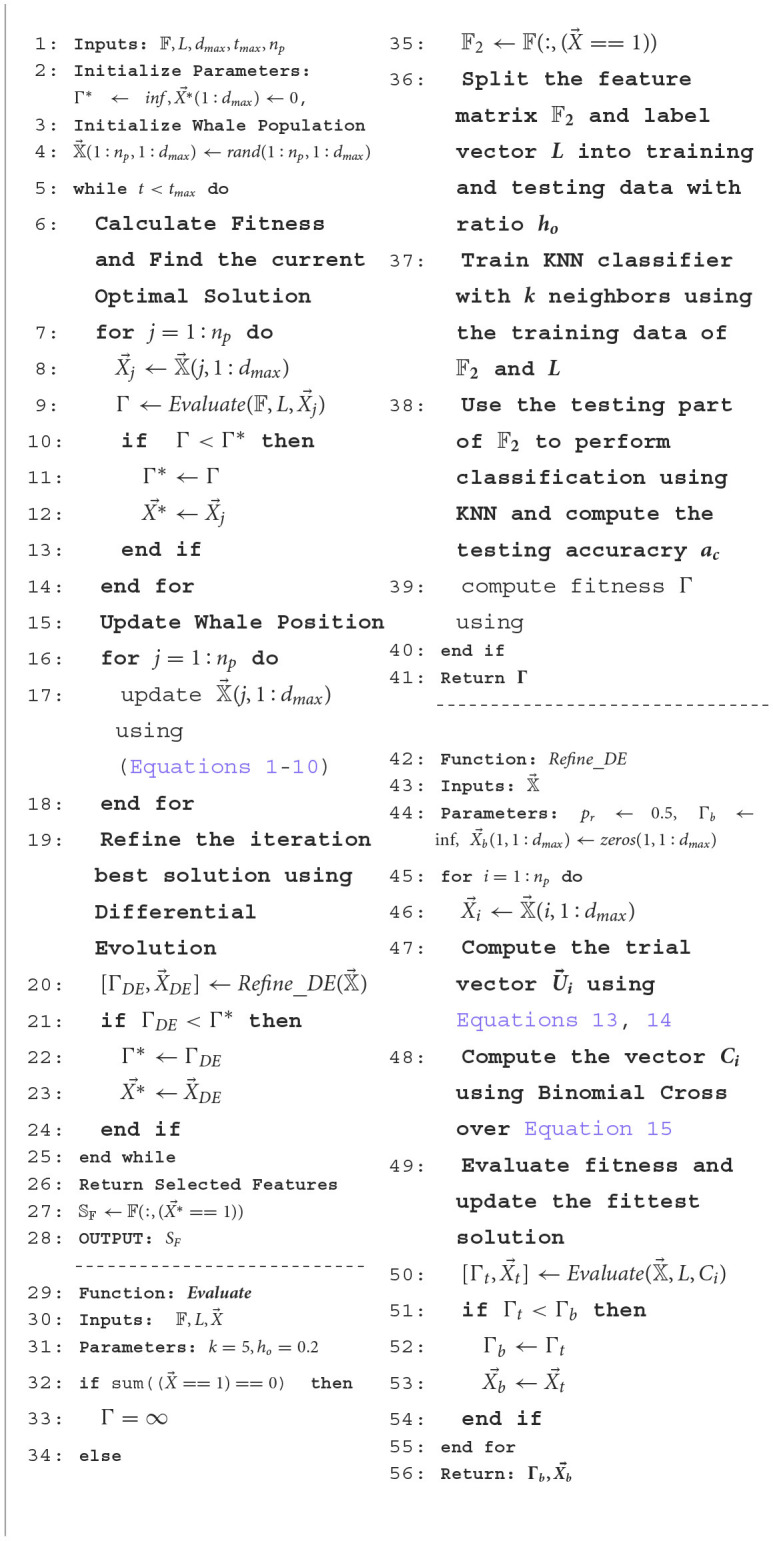
Proposed hybrid BWO based feature selection algorithm.

**Table 4:** Description of main symbols used in Algorithm 1.

**Symbol**	**Description**	**Symbol**	**Description**
𝔽	Fused feature matrix	L	Label vector of training set
*d*_*max*_	Total no. of fused features per image	*t*_*max*_	maximum no. of hybrid BWO algorithm
*n*_*p*_	Population size	$\vec{𝕏}$	Whale population matrix
$\vec{X^{*}}$	Iteration best individual	Γ^*^	Iteration best fitness
$\vec{X}$	One binary individual of population matrix $\vec{𝕏}$	𝔽_2_	Features extracted from 𝔽
*h*_0_	split ratio of training and texting parts of 𝔽 for KNN	K	size of neighbors for KNN
*a*_*c*_	classification accuracy	Γ	fitness value (error rate)
*n*_*pred*_	No. of successfully predicted labels	*n*_*test*_	total no. of test labels
*p*_*r*_	Binomial crossover probability differential evolution	${\vec{M}}_{i}$	Mutation vector
${\vec{U}}_{i}$	Trial vector	${\vec{C}}_{i,j}$	Binomial crossover vector

In Steps 15–18 of the main routine, the fittest solution (leader whale) is used to update the position of all other whales of population $\vec{𝕏}$ using the update rules (Equations 1–10). The updated whales population is given as an input to *Refine*_*DE* which performs refinement of best solution using differential evolution. If a better solution is obtained by performing mutation and crossover rules (Equations 13–15) of DE, this solution is selected as the iteration best of BWO algorithm. At the conclusion of *t*_*max*_ iterations of the BWO algorithm, Step 27 involves utilizing the indices of non-zero entries in the best overall solution $\vec{X^{*}}$, to choose the corresponding features from the set 𝔽.

#### 3.2.5 Classification

The ensemble of selected features yielded by the proposed hybrid BWO algorithm, in conjunction with the label vector *L*, is subsequently employed for training the outer classifiers. In this study, we conducted an assessment of the classification efficacy across a spectrum of classifiers employing diverse kernel configurations, ultimately identifying and adopting the top-performing classifiers for our proposed study.

## 4 Performance results

The prescribed workflow for the detection and sub-type categorization of acute lymphoblastic leukemia has been executed using MATLAB R2021a, running on an Intel Core i7 CPU equipped with 16GB of RAM, all hosted within a 64-bit Windows 10 operating environment.

### 4.1 Leukemia binary detection

In the first phase, the leukemia detection pipeline is applied to the ALL-IDB2 dataset. To mitigate potential overfitting issues, the pre-processed images within the dataset undergo an augmentation procedure. This step involves random image rotations within the range of [0, 360] degrees, resizing by a random factor within [0.5, 1] interval. The distribution of images across various classes of augmented ALL-IDB2 dataset is presented in Table 5. Next, the augmented dataset was stratified into training and validation sets with a 70:30 ratio through a random selection of images belonging to each class. The corresponding image distribution is shown in Table 6.

**Table 5:** Class-wise image details of augmented ALL-IDB2 dataset.

**Class type**	**No. of images**	
	**Original dataset**	**Augmented dataset**
Healthy subject	130	600
ALL affected subject	130	590

**Table 6:** Distribution of ALL-IDB2 dataset into training and test parts.

**Class**	**Training dataset**	**Testing dataset**
Healthy subject	420	180
ALL affected subject	413	177
Total No. of images	833	357

**Figure 5:**
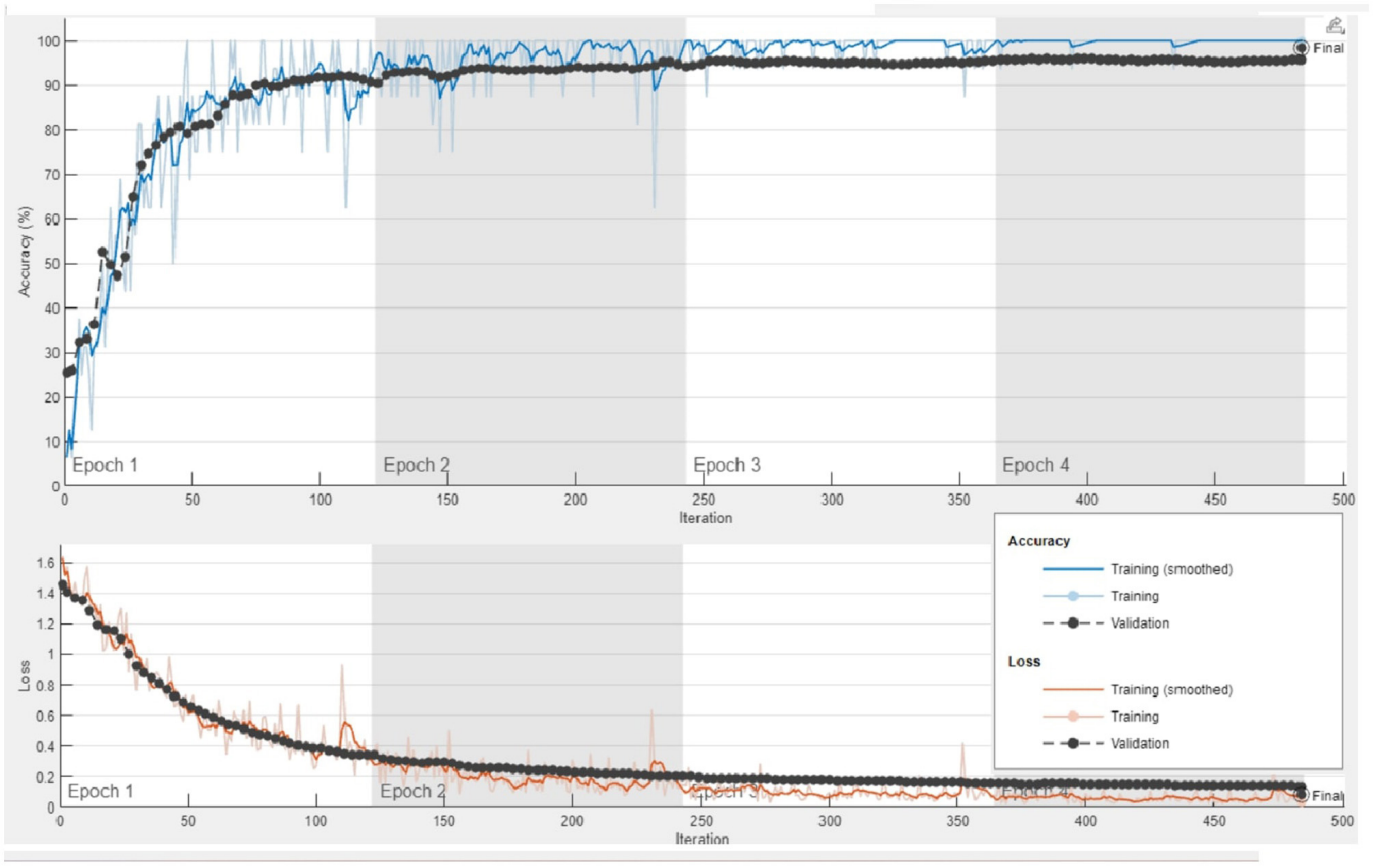
Plots of training accuracy and loss function for transfer learning of proposed custom CNN model on augmented ALL-IDB2 dataset.

To perform feature extraction, the training dataset is employed for transfer learning with both the GoogleNet model and our proposed custom CNN architecture. The main training parameters are shown in Table 7. We explored various combinations of hyperparameters through multiple training runs and identified the set that achieved the best training performance. These optimal parameters were then used to train the custom CNN on the augmented ALL-IDB2 dataset. Figure 5 shows the validation accuracy and loss function plot of proposed custom CNN on the augmented ALL-IDB2 dataset. Subsequently, deep feature vectors of dimensions 1, 024 and 2, 048 are, respectively, extracted from GoogleNet and custom CNN. These feature vectors are then horizontally concatenated, yielding a composite feature vector of size 1 × 3, 072 for each training image. In the next step, the proposed hybrid BWO algorithm is applied on fused feature vector for the selection of most dominant set of features. The vector of selected features is then used for training outer classifiers. In this study, we have used a range of classifier families, such as SVM, KNN, NN, Decision Tree (DT), and Ensemble, with different kernel settings. The performance results of best performing classifiers from each family are shown in Table 8. The key performance metrics are evaluated, which include classification Accuracy, Precision, Sensitivity (Recall), F1 Score, and Specificity. For binary classification, these metrics are computed as Equations 18–22:


(18) $$\begin{array}{c} \text{Accuracy}=\frac{TP}{TP+TN+FN+FP} \end{array}$$

(19) $$\begin{array}{c} \text{Sensitivity (Recall)}=\frac{TP}{TP+FN} \end{array}$$

(20) $$\begin{array}{c} \text{Specificity}=\frac{TN}{TN+FP} \end{array}$$

(21) $$\begin{array}{c} \text{F1 Score}=\frac{2\times \text{Precision}\times \text{Recall}}{\text{Precision}+\text{Recall}} \end{array}$$

(22) $$\begin{array}{c} \text{Precision}=\frac{TP}{TP+FP} \end{array}$$

where *TP* denotes the total number of “ALL” images successfully classified, *TN* denotes the total number of “Healthy” images classified as “Healthy,” *FP* denotes the number of ‘Healthy' images incorrectly classified as “ALL,” and *FN* denotes the number of “ALL” images incorrectly classified as “Healthy.”

**Table 7:** Main parameters for transfer learning of GoogleNet and proposed custom CNN model.

**Parameter**	**Value**	**Parameter**	**Value**
Kernel type	sdgm	Max epochs	10
Initial learning rate	1 × 10^−4^	Environment	Auto
Validation frequency	30	Stride size	1
Mini batch size	20	Dropout rate	0.1

**Table 8:** Performance metrics of leukemia binary detection on ALL-IDB2 dataset.

**Classifier**	**Kernel**	***N*_*t*_**	***N*_*s*_**	**Accuracy**	**Precision**	**Sensitivity**	**F1 Score**	**Specificity**
KNN	Cosine	3,072	460	93.2773	0.9000	0.9643	0.9048	0.9310
	Coarse			94.6218	0.9000	0.9926	0.9071	0.9441
	Cubic			94.1176	0.9167	0.9649	0.9194	0.9402
SVM	Gaussian			92.8571	0.9000	0.9558	0.9040	0.9270
	Regression			94.1176	0.9250	0.9569	0.9262	0.9407
	Quadratic			94.7899	0.9333	0.9622	0.9342	0.9475
Decision Tree	Medium			89.0756	0.8500	0.9273	0.8594	0.8870
NN	Narrow			91.5966	0.8667	0.9630	0.8769	0.9123
	Wide			94.2017	0.8917	0.9926	0.9002	0.9394
Ensemble	Rusboost			98.4034	0.9750	0.9932	0.9750	0.9840
	Subspace KNN			99.1597	0.9944	0.9888	0.9944	0.9915

*N*_*t*_: total no. of features in fused feature set, *N*_*s*_: no. of features selected by hybrid BWO algorithm.

**Figure 6:**
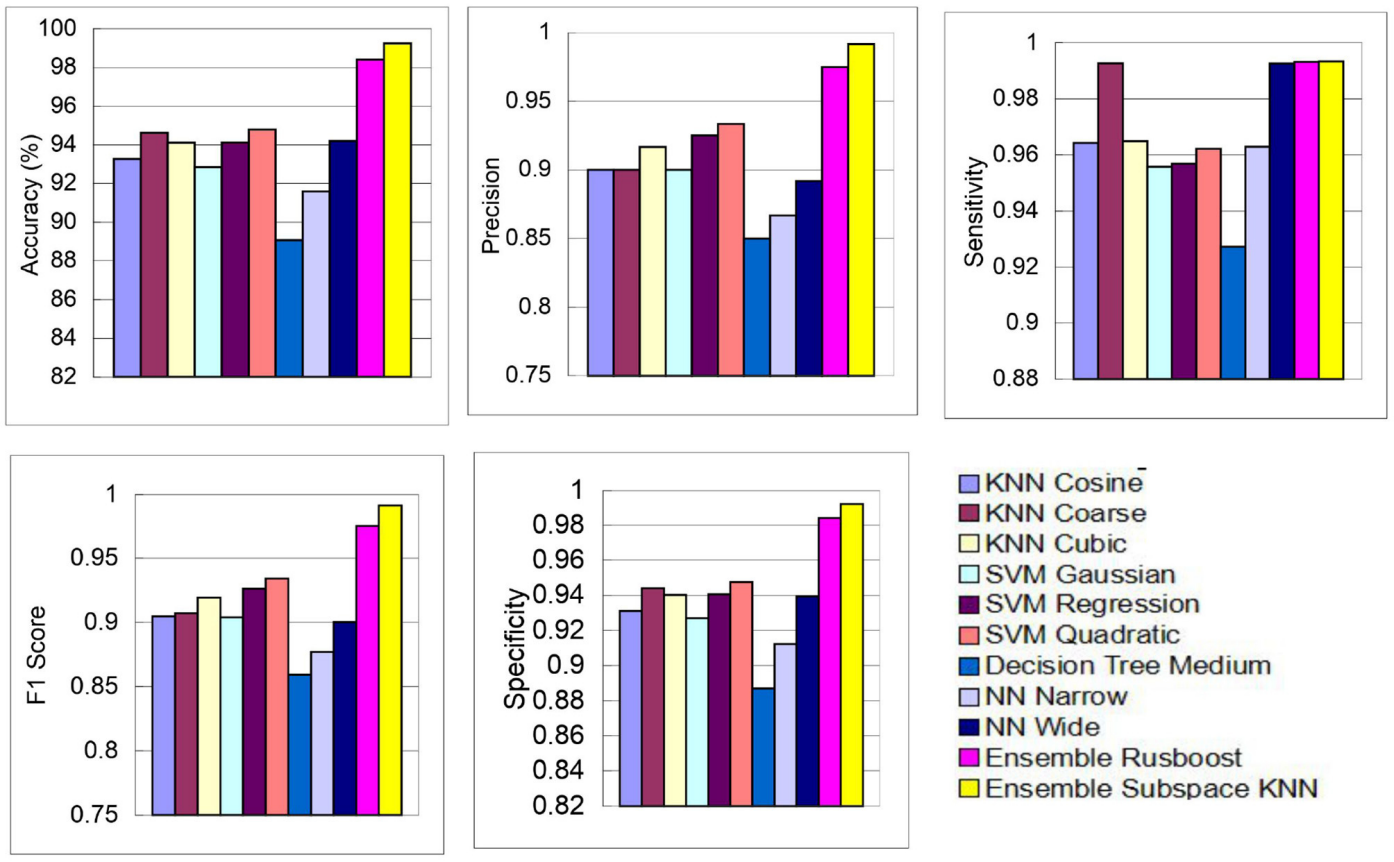
Graphical representation of performance results of proposed pipeline for leukemia detection using ALL-IDB2 dataset.

The above performance metrics reported in Table 8 are the average results obtained after several Monte-Carlo iterations of proposed pipeline with 10-fold cross validation. In Figure 6, the individual results of each classifier are graphically presented for comparison. Out of 3, 072 features extracted from transfer learning of GoogleNet and proposed custom CNN, only 460 features are selected by Hybrid BWO algorithm. With an 85% feature reduction, all selected classifiers demonstrate accuracy above 89%. The Ensemble Subspace KNN classifier demonstrates an average accuracy of 99.2% and better or comparable values of key performance parameters in comparison with other classifier settings.

**Figure 7:**
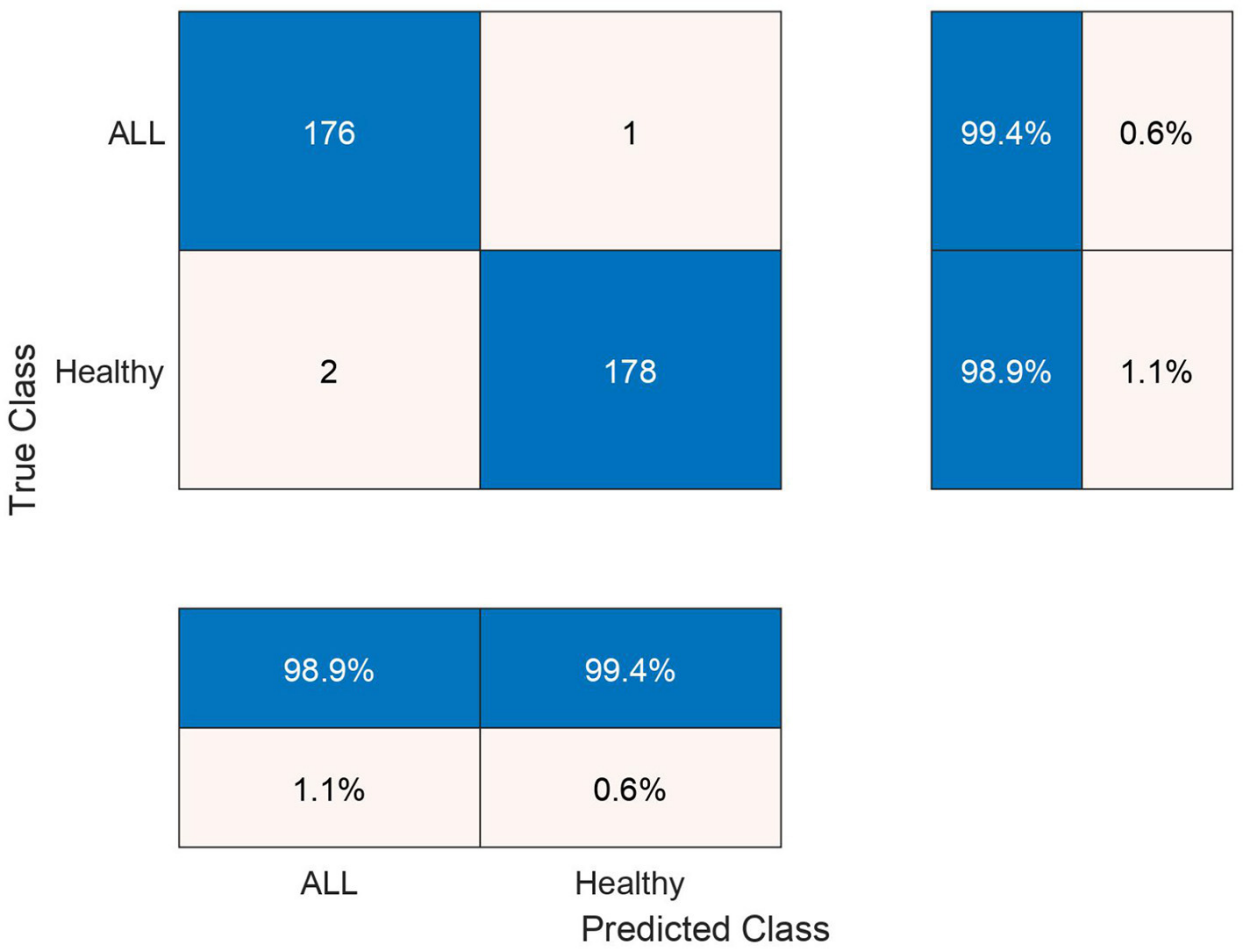
Testing confusion matrix of ensemble subspace KNN classifier on ALL-IDB2 dataset.

**Figure 8:**
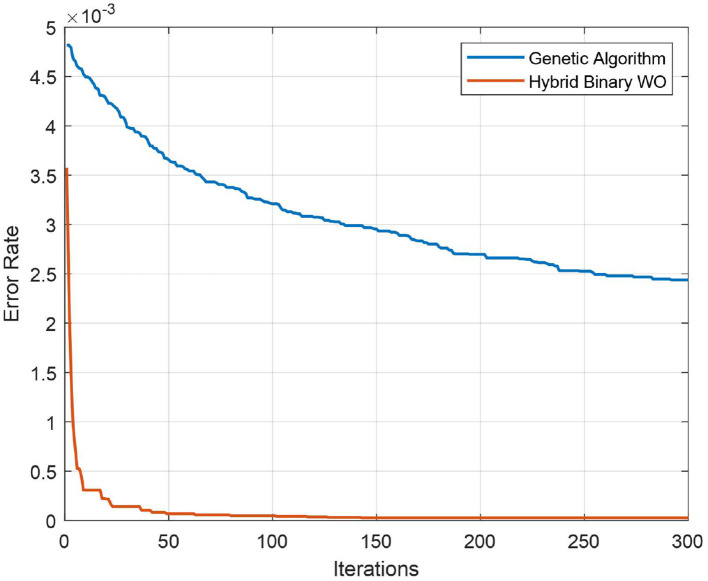
Convergence plot of proposed hybrid binary whale optimization and genetic algorithm.

The test confusion matrix of Ensemble Subspace KNN classifier on ALL-IDB2 dataset is shown in Figure 7, which indicates a high true positive rate (TPR) and a very low false negative rate (FNR), confirming the accuracy of our method. Furthermore, in Figure 8, the error rate of feature selector using proposed hybrid BWO algorithm is plotted with classical Genetic Algorithm (GA). The error rate Γ is computed using the Equation 17. Both GA and BWO are population-based search algorithms, the hybrid BWO demonstrates a better exploration of search space by achieving significantly smaller error rate for all iterations.

**Figure 9:**
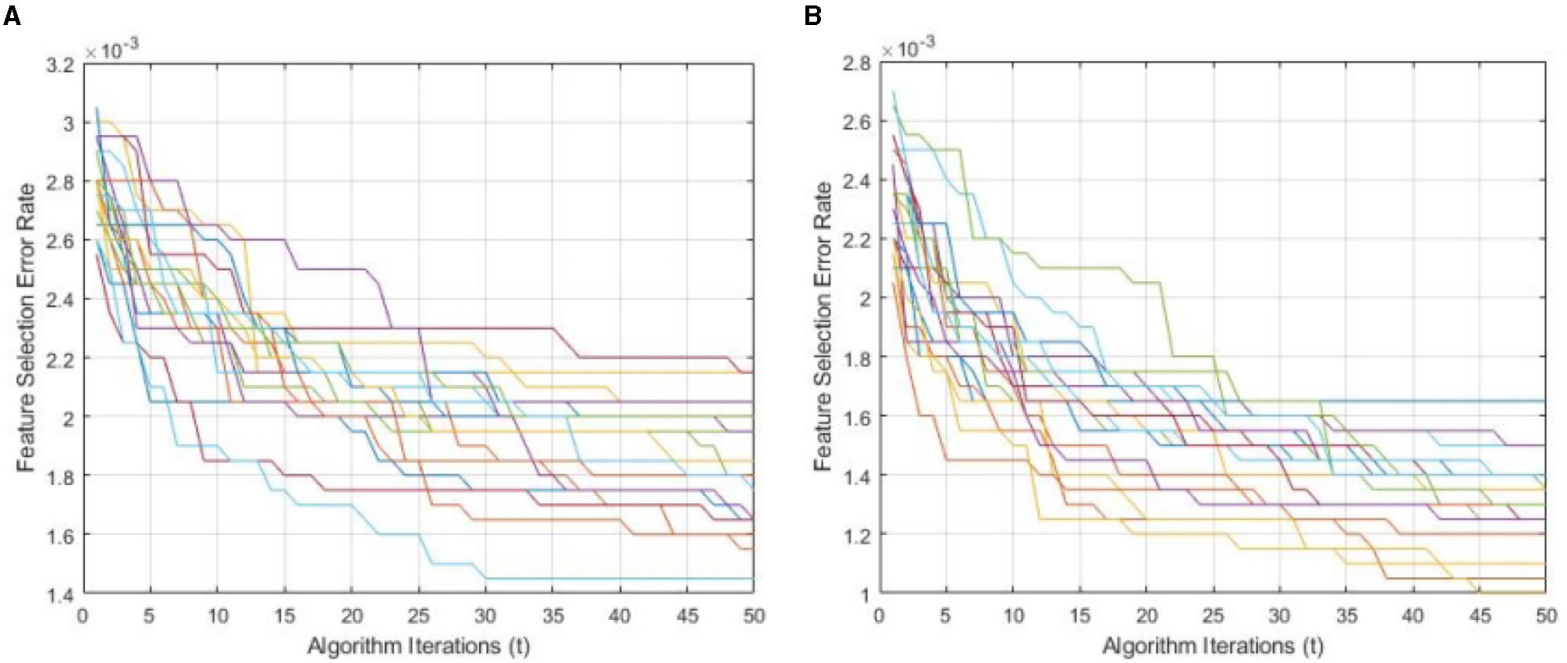
Convergence performance of standard and proposed hybrid BWO algorithm ON ALL-IDB2 dataset. **(A)** Standard BWO algorithm. **(B)** Proposed memetic BWO algorithm.

**Figure 10:**
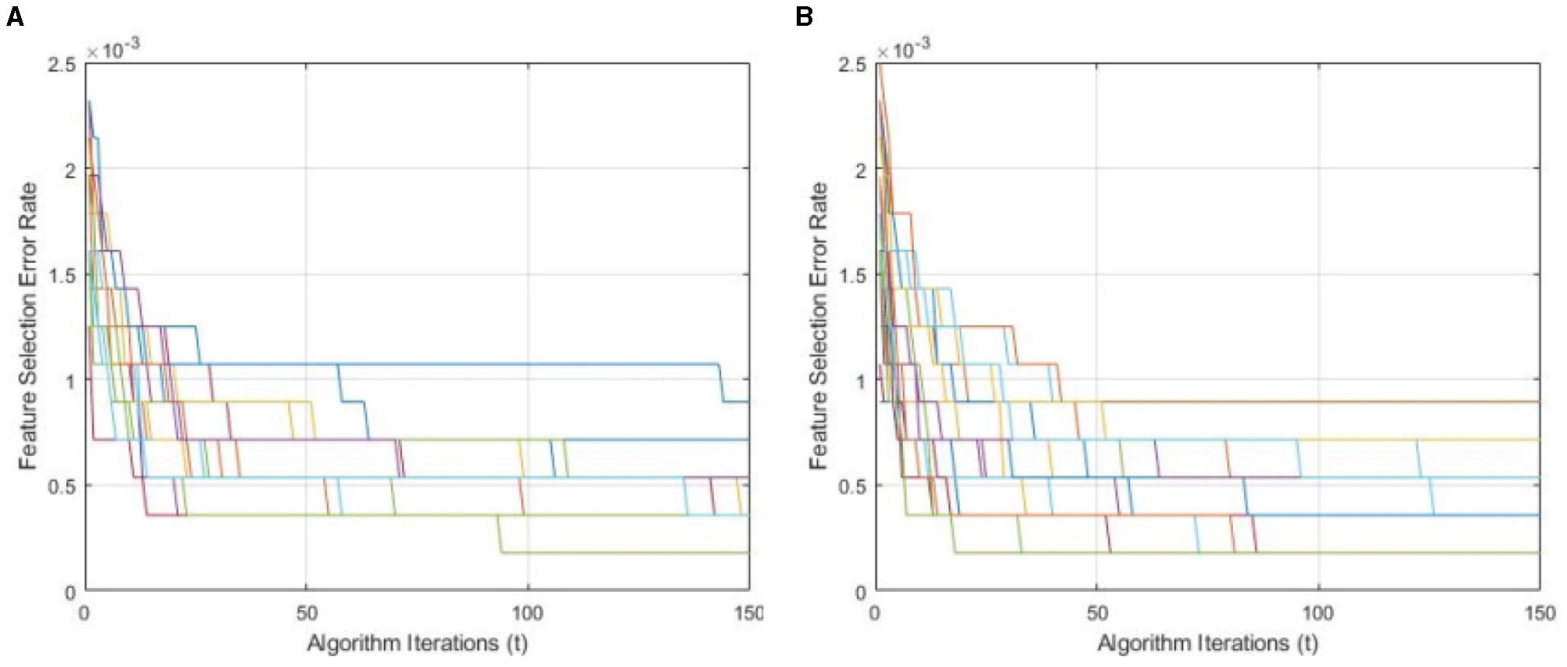
Convergence performance of standard and proposed hybrid BWO algorithm on multi-class dataset of the study mentioned in the reference Ghaderzadeh et al. (2022). **(A)** Standard BWO algorithm. **(B)** Proposed memetic BWO algorithm.

Figure 9 demonstrates a performance comparison of standard BWO and proposed hybrid BWO algorithms. The graphs in the figure are generated by performing several Monte Carlo iterations of both algorithms on the same training and testing portions of ALL-IDB2 dataset and other common parameters. Each curve in the graph is obtained for one Monte Carlo iteration of the corresponding algorithm and plots the error rate as a function of *t* iterations (generations) of the algorithm. Each algorithm runs for *t*_*max*_ = 50 times per Monte Carlo iteration. The graphs clearly reveal a better convergence performance of proposed hybrid BWO algorithm with DE-based local search method. For example, for *t* = 50, the best error rate achieved by standard BWO is 1.5 × 10^−3^, whereas, for the same value of *t*, proposed hybrid BWO achieves an error rate of 1.0 × 10^−3^, which is ~30% smaller as compared with the standard BWO algorithm. This shows the superiority of proposed local search-based solution refinement strategy. In Figure 10, the convergence performance of two algorithms is plotted for multi-class dataset of the study mentioned in the reference Ghaderzadeh et al. (2022). The graphs again reveal a faster convergence rate of proposed hybrid BWO algorithm as compared with its standard version.

### 4.2 Leukemia sub type identification

**Figure 11:**
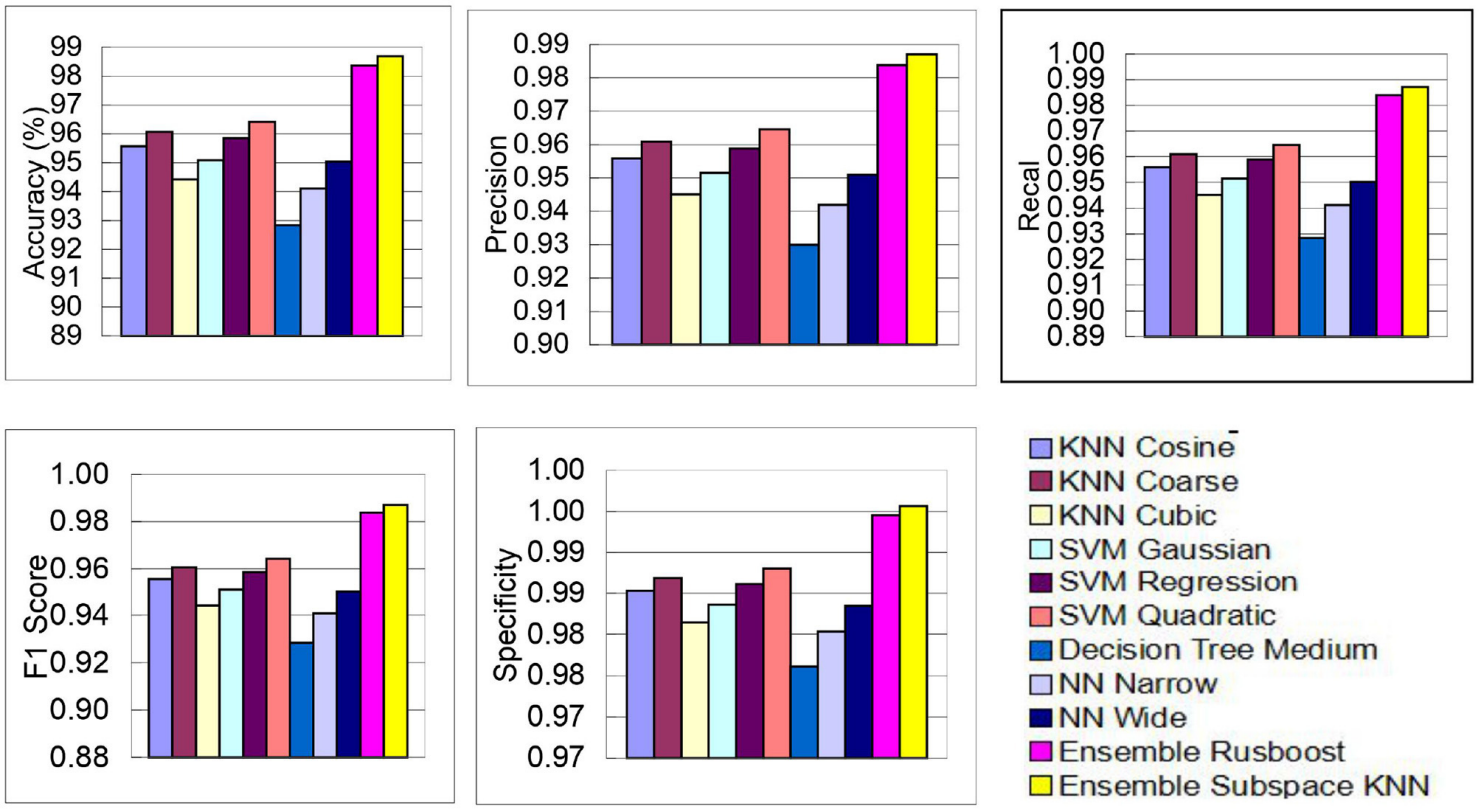
Performance results of selected classifiers for ALL sub type classification using dataset of the study mentioned in the reference Ghaderzadeh et al. (2022).

**Figure 12:**
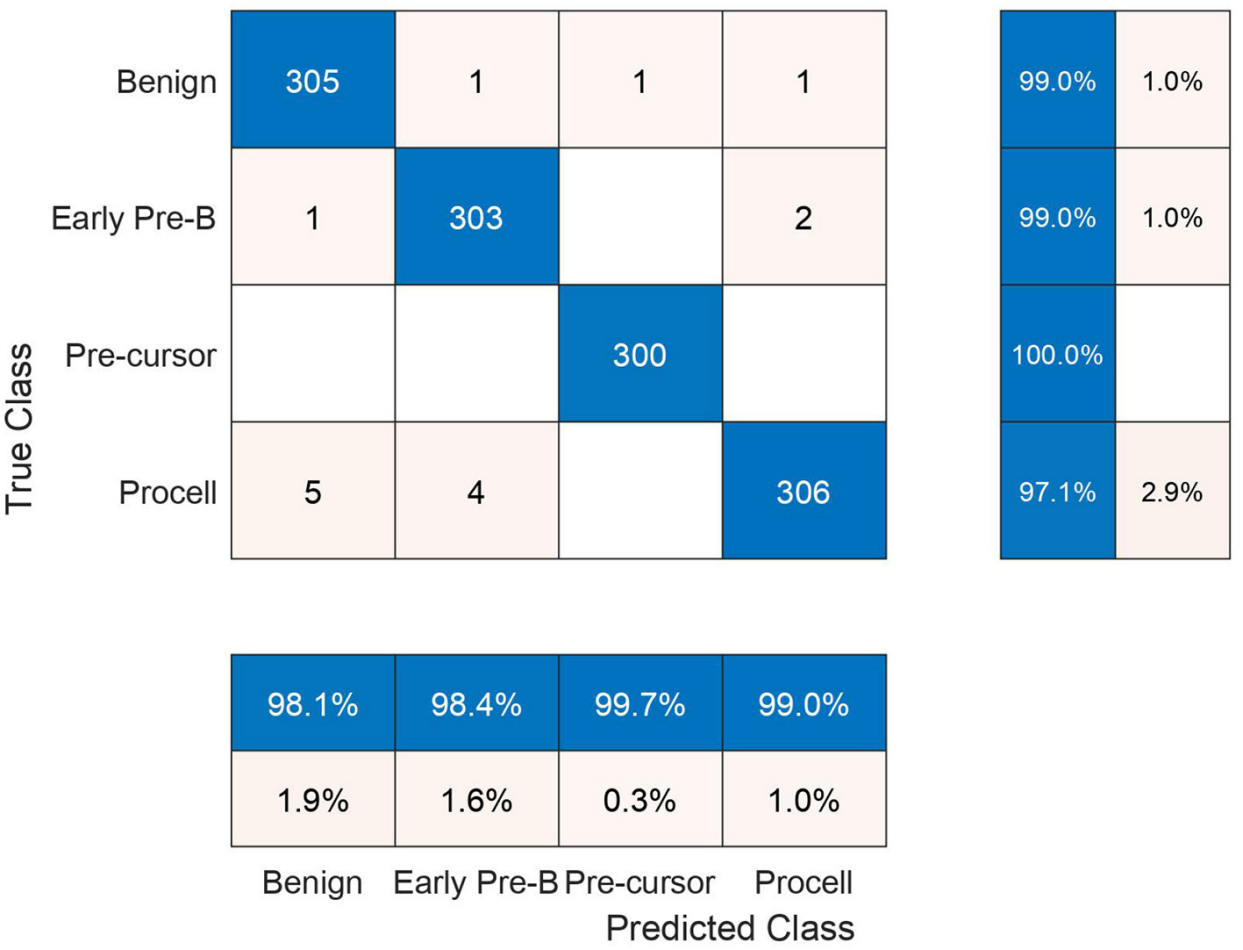
Testing confusion matrix of ensemble subspace KNN classifier on dataset of the study mentioned in the reference Ghaderzadeh et al. (2022).

In the subsequent stage, the proposed pipeline is employed for the purpose of leukemia sub type classification, utilizing the dataset of the study mentioned in the reference Ghaderzadeh et al. (2022). Dataset diversity is augmented through randomized rotation and scaling of images along with the application of color jitter. The image distribution of augmented dataset is shown in Table 9. In Table 10, the class distribution of images is demonstrated after performing random splitting of augmented dataset into training and validation parts with a 70:30 ratio. Using the similar approach of binary classification, the training dataset is used for transfer learning of GoogleNet and proposed custom CNN. The features are extracted from both networks and concatenated together to obtain a fused feature vector. The set of selected features is then obtained using proposed hybrid BWO algorithm and subsequently used for training of outer classifiers. Table 11 shows the ALL multi-class identification performance of selected classifiers. The average results are computed from several Monte-Carlo iterations of proposed pipeline. During each iteration, the overall value of all performance metrics of Table 11 is computed by micro-averaging of their individual class-wise values. A comparison of individual metrics of all classifiers is presented in Figure 11. Again, a better performance is demonstrated by the Ensemble Subspace KNN, which obtains an overall average accuracy of 98.6981% with relatively good values of other performance metrics. The confusion matrix of Ensemble Subspace KNN is shown in Figure 12.

**Table 9:** Distribution of augmented dataset of the study mentioned in the reference Ghaderzadeh et al. (2022).

**Class**	**Frequency**	
	**Before**	**After**
Benign	512	1,024
Pre-cursor	955	1,000
Pro-cell	796	1,050
Early-pre-B	979	1,020

**Table 10:** Class distribution of training and testing parts of dataset of the study mentioned in the reference Ghaderzadeh et al. (2022) for leukemia sub type classification.

**Class**	**Training images**	**Testing images**	**Total**
Benign	716	308	1,024
Pre-cursor	700	300	1,000
Pro-cell	735	315	1,050
Early pre-B	714	306	1,020
Total	2,149	1,229	4,094

**Table 11:** Performance metrics of leukemia sub type classification using dataset of the study mentioned in the reference Ghaderzadeh et al. (2022).

**Classifier**	**Kernel**	***N*_*t*_**	***N*_*s*_**	**Accuracy**	**Precision**	**Sensitivity**	**F1 Score**	**Specificity**
KNN	Cosine	3,072	460	95.5701	0.9558	0.9560	0.9557	0.9853
	Coarse			96.0623	0.9608	0.9610	0.9606	0.9869
	Cubic			94.4217	0.9451	0.9451	0.9442	0.9815
SVM	Gaussian			95.0779	0.9515	0.9515	0.9509	0.9836
	Regression			95.8503	0.9589	0.9589	0.9585	0.9862
	Quadratic			96.4199	0.9645	0.9646	0.9642	0.9881
Decision tree	Medium			92.8397	0.9299	0.9284	0.9283	0.9761
NN	Narrow			94.1128	0.9419	0.9412	0.9409	0.9804
	Wide			95.0366	0.9510	0.9503	0.9501	0.9834
Ensemble	Rusboost			98.3673	0.9837	0.9839	0.9837	0.9946
	Subspace KNN			98.6981	0.9870	0.9872	0.9871	0.9957

Table 12 provides a comparison between the performance of our proposed approach and several existing studies focused on leukemia identification. To ensure a fair assessment, we specifically selected previously published studies that utilized either identical or highly similar datasets. Our proposed pipeline, designed for both binary leukemia detection and sub-type identification, demonstrates superior or at least comparable performance metrics compared with various other relevant investigations that employed smaller feature sets. These results affirm the effectiveness and practicality of our proposed methodology.

**Table 12:** Comparison of classification accuracy of proposed leukemia identification pipeline with some existing relevant studies.

**Work**	**Methodology**	**Data set**	**Classification type**	**Performance results**
Di Ruberto et al. (2020)	Classification: SVM, ANN Multi scale blop detection Deep feature extraction: AlexNet Classification: SVM	ALL-IDB	Binary classification of ALL	Accuracy = 94.1%
Bodzas et al. (2020)	Preprocessing Segmentation: three phase filtering Morphological feature extraction	Self collected	Binary classification of ALL	Specificity = 93.5%
Hegde et al. (2020)	Active contours for nuclei detection Shape and texture features extraction Classification: NN, SVM	Self collected	Leukemia binary detection	Accuracy = 98.8%
Baig et al. (2022)	Preprocessing Feature extraction: hybrid CNN Classification: bagging ensemble	ALL-IDB MiMMSBI SN-AM	ALL binary classification AML binary classification Multiple myeloma binary classification	ALL Classification Accuracy = 97.04%
This work	Contrast stretching using DE Deep feature extraction: proposed custom CNN & GoogleNet Feature selection: hybrid BWO algorithm	ALL-IDB2 Ghaderzadeh et al. (2022)	ALL binary classification ALL sub-type classification	Accuracy = 99.15% Accuracy = 98.69%

### 4.3 Statistical analysis

In this study, we applied the one-way analysis of variance (ANOVA) (Fotso Kamga et al., 2018) method to verify the validity of classification results from statistical point of view. The statistical analysis was performed on classification accuracy as the key performance metric. For this purpose, a number of Monte Carlo iterations of the complete classification pipeline were performed with 10 fold-cross validation in each iteration. The accuracy values were collected for the above mentioned classifiers. The normality of accuracy data was validated using Shapiro–Wilk test (Akram et al., 2020). The homogeneity of variances of classifier accuracy values were verified using Bartlet's test (Ahmad et al., 2023a). The significance level α = 0.05 was selected. The *p*-values of KNN, SVM, Decision Tree, NN, and Ensemble family of classifier were *p*_1_ = 0.723, *p*_2_ = 0.7021, *p*_3_ = 0.694, *p*_4_ = 0.660 and *p*_5_ = 0.651, respectively, along with chi-squared probability *p*_*ch*_ = 0.825. The obtained p-values were less than α, which confirmed that null hypotheses of Shapiro–Wilk and Bartlet's test are true, i.e., accuracy values are normally distributed with homogeneous variances.

Table 13 shows the results of one-way ANOVA test performed on accuracy of selected classifiers. The key metrics include mean square error (MSE), degree of freedom (df), *F*-statistics, *p*-value, and sum of square deviation (SS).

**Table 13:** ANOVA statistical results of proposed pipeline.

**V-source**	**df**	**SS**	**MSE**	***F*-statistics**	***p*-value**
Between	2	6.815 × 10^−5^	2.7347 × 10^−5^	0.29	0.695
Within	6	6.3245 × 10^−4^	8.7725 × 10^−5^	–	–
Total	8	5.956 × 10^−4^	–	–	–

The confidence interval plot of selected classifiers on the proposed leukemia identification pipeline is shown. The average accuracy is demonstrated as red line, whereas the 95% confidence limits are shown as black lines. The figure demonstrates that ensemble subspace achieves a high average accuracy with small confidence interval as compared with other classifiers. The upper and lower quantile points of each classifier lie within the confidence interval limits.

## 5 Discussion

In this study, we examined the effectiveness of our proposed approach for the binary and multi-class identification of ALL. Modern deep CNNs often come with large model sizes, demanding significant memory and computational resources. Employing an ensemble of networks, including a tailored CNN alongside publicly available deep CNN models, offers a practical compromise between classification performance and pipeline complexity. Furthermore, leveraging pretrained CNN models for feature extraction and employing external classifiers is a potent and pragmatic strategy that amalgamates the advantages of transfer learning, feature abstraction, and minimized training effort to enhance the outcomes of diverse computer vision tasks. One drawback of this approach is that the feature sets extracted from deep CNNs often exhibit substantial size and encompass a considerable amount of duplicate features. Selection of most promising set of features is a combinatorial optimization problem with computational complexity of exhaustive search growing exponential with the size of feature vector. Population-based feature selection methods have shown a significant research interest in recent years. A number of bio-inspired and nature-inspired meta-heuristics have been proposed. One challenge is the exploration and exploitation capabilities of the algorithm problem of local optima. To address this issue, we have proposed a memetic feature selection approach that combines elements of population-based algorithms with local search methods. In particular, we have proposed a nature-inspired metaheuristic name binary whale optimization algorithm in which optimization of an iteration best solution is performed using a differential evolution method. These optimizations at CNN architecture and feature selection level yield an improved pipeline which shows promising results for leukemia detection and sub-type classification. The validity of proposed approach is manifested with better performance results as compared with several recently published studies.

## 6 Conclusion

Leukemia, a hematologic malignancy, afflicts both pediatric and geriatric populations. Acute lymphoblastic leukemia is an aggressive form of leukemia that has a high mortality rate. Modern computer vision approaches and deep CNNs have been demonstrated as potential solutions for computer aided diagnosis of several medical conditions. However, precise classification of malignancies at microscopic level is a challenging task due to morphological similarities between different blood entities. This study presents an improved pipeline for enhancing leukemia detection from blood smear images. At first, We propose an intricately designed 88-layer deep CNN architecture inspired by AlexNet and SqueezeNet. We used this network as a feature extractor alongside GoogleNet, aiming to balance classification accuracy and computational efficiency. The work then models the feature selection problem as a combinatorial optimization problem and proposes a novel memetic approach based on the Hybrid binary whale optimization algorithm to meticulously select the most dominant set of features. Our proposed methodology undergoes rigorous validation using publicly available datasets containing peripheral blood smear images across diverse leukemia classes. The proposed feature selection approach effectively selects the most dominant and discriminant set of features. The proposed system achieves an overall accuracy rate of 99.15% with an 80% reduction in feature size, performing comparably or better than several existing studies on leukemia identification. The propose method can be extended to the diagnosis of other blood-related diseases. It can complement advanced diagnostic methods such as RNA sequencing and molecular testing by providing additional supporting evidence. Additionally, it offers smooth integration with practical image analysis systems such as image flow cytometry, expanding their functionalities in real-world settings.

## Data availability statement

Publicly available datasets were analyzed in this study. This data can be found here: Scotti et al. (2005) and Ghaderzadeh et al. (2022).

## Author contributions

MA: Conceptualization, Data curation, Investigation, Methodology, Software, Validation, Writing – original draft, Writing – review & editing. RA: Methodology, Software, Validation, Writing – original draft. NK: Supervision, Writing – review & editing. AA: Writing – review & editing. NA: Writing – review & editing. AM: Resources, Validation, Writing – review & editing.

## Footnotes

^1^ http://www.image-net.org
